# Left Isomerism With Normal Bronchopulmonary Anatomy: Broadening the Heterotaxy Spectrum

**DOI:** 10.1155/crra/5512404

**Published:** 2025-04-17

**Authors:** Zach Sukin, Erin Moffett, Madison Wulfeck, Dennis Lindfors, Sandor Szilagyi

**Affiliations:** Department of Radiology at HCA Healthcare/USF, Morsani College of Medicine, GME/HCA Florida Trinity Hospital, Florida, USA

## Abstract

Situs ambiguous is a rare congenital condition characterized by the abnormal arrangement of thoracoabdominal organs along the left–right axis. This condition often presents as either left or right isomerism, leading to complex anatomical variations and associated clinical challenges. We present the case of a 44-year-old female who was incidentally discovered to have situs ambiguous with polysplenia and left atrial appendage isomerism during the evaluation of abdominal pain and urinary symptoms caused by a ureteral calculus. Notably, the patient exhibited normal bronchopulmonary anatomy. The patient underwent a ureteroscopy, laser lithotripsy, stone extraction, and right ureteral stent placement. The patient was discharged shortly thereafter. We believe our case underscores the critical importance of recognizing the potential dissociation between thoracic and abdominal isomerism. It also highlights the need for further investigation into the embryological processes that contribute to these unusual presentations.

## 1. Introduction

Situs ambiguous, also known as heterotaxy, is a congenital condition characterized by abnormal alignment of the thoracoabdominal organs along the left–right (L-R) axis. This definition implies that the alignment differs from normal organ arrangement (situs solitus) and mirror-image organ arrangement (situs inversus), as situs ambiguous involves organs in nonstandard patterns. It is an uncommon disorder with an estimated prevalence of about 0.0001% [1]. Situs ambiguous can be further classified into polysplenia syndrome or asplenia syndrome based on the presence or absence of multiple spleens, respectively.

Isomerism refers to birth defects affecting thoracic structures, where paired structures on opposite sides of the (L-R) axis are symmetrical or nearly symmetrical. In left isomerism, paired structures have the morphological appearance of normal left-sided structures, manifesting as bilateral hyparterial upper lobe bronchi, bilateral bilobed lungs, and isomeric left atrial appendages. Left isomerism is typically associated with abdominal heterotaxy with polysplenia. In contrast, right isomerism manifests as bilateral eparterial upper lobe bronchi, bilateral trilobed lungs, and isomeric right atrial appendages. Right isomerism is typically associated with abdominal heterotaxy with asplenia [2]. However, such cases are frequently associated with complex congenital malformations, including congenital heart disorders, leading to higher morbidity and mortality rates. Heart defects in children account for significant mortality, with up to 90% of children diagnosed with right isomerism dying at birth [3].

Here, we present a case in which dissociation between thoracic and abdominal isomerism was observed, as the patient exhibited normal bronchopulmonary anatomy despite having polysplenia and left atrial appendage isomerism [2, 3]. Yim et al. studied 110 patients with definable anatomy and found that 63 (57%) had polysplenia, with 89% having multiple spleens and 11% having a single lobulated or round spleen. The majority (75%) had a left-sided bronchopulmonary branching pattern, while 8% had a right-sided pattern. Notably, only 11% had normal pulmonary anatomy, highlighting the rarity of this finding in patients with polysplenia. Such cases provide a unique opportunity to further understand the spectrum of polysplenia and its relationship to thoracic anatomy [4].

L-R laterality genes, including ZIC3, NODAL, LEFTY2, CFC1, and SHROOM3, play a role in causing heterotaxy [5]. In cases of situs ambiguous with polysplenia and left isomerism, the differential presentation of thoracic and abdominal organs can be explained by their distinct embryological developmental processes and specific genetic abnormalities. Both the heart and lungs arise from multipotent cardiopulmonary progenitors (CPPs). Disruptions in Nodal signaling affect these progenitors, leading to changes in both organs [6].

In rare cases where the lungs remain unaffected despite abnormalities in the heart and abdomen, a compensatory mechanism or redundancy in the signaling pathways specific to lung development may be responsible. This redundancy may not be present in cardiac or abdominal development pathways, making them more susceptible to disruptions [6, 7].

Situs ambiguous is typically linked to disruptions in genetic pathways crucial for the development and positioning of abdominal organs. The Nodal pathway, which directs left-side specification in the embryo, plays an essential role in maintaining proper organ positioning [8]. PITX2, a transcription factor upregulated on the left side of the body by Nodal proteins, is critical for the asymmetric organogenesis of the heart and other visceral structures [9, 10]. Mutations in PITX2 or its regulatory elements may not uniformly affect all L-R signaling pathways. The heart, for example, is particularly sensitive to PITX2 levels, showing more pronounced effects from mutations or disruptions compared to the lungs [11]. LEFTY and ZIC3 help ensure that Nodal signaling remains correctly localized, and their dysfunction can lead to defects in organ placement.

Fluid movement generated by motile cilia aids in organ arrangement by activating the Nodal pathway on the left side of the embryo [9]. Heterotaxy syndromes can result from mutations in ciliary function, the Nodal pathway, or ZIC3, leading to interference with L-R asymmetry, which is essential for organ positioning [8, 9, 12]. Consequently, disruptions in these pathways affect the positioning of the liver, spleen, and other abdominal organs, leading to polysplenia and left isomerism [13].

The dissociation between thoracic and abdominal development is uncommon, as the same genetic abnormalities that cause misplacement in one region typically affect the other. Such cases challenge conventional wisdom, as L-R patterning genes usually exert systemic effects, leading to multiple organ displacements when disrupted [14–16]. Therefore, our case, in which pulmonary structures developed normally while cardiac and abdominal organs were abnormal, challenges current understanding and highlights the complexity of genetic regulation during embryogenesis [16]. These anomalies underscore the need for further research to identify the specific genetic and molecular mechanisms that allow for such developmental dissociation.

This case report not only challenges our understanding of organ development in situs ambiguous but also highlights the importance of recognizing atypical presentations. Given the complexities associated with heterotaxy syndromes and their potential complications, early diagnosis is crucial. Such cases suggest that additional embryological factors may be at play, warranting further study. Although rare, comparative studies of atypical heterotaxy presentations provide valuable insights into the variability of organ development.

## 2. Case Presentation

**Figure 1:**
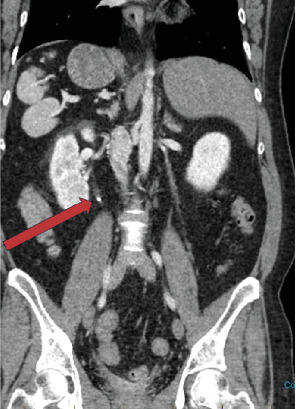
Coronal noncontrast CT demonstrates kidney stone in the right ureter (red arrow).

**Figure 2:**
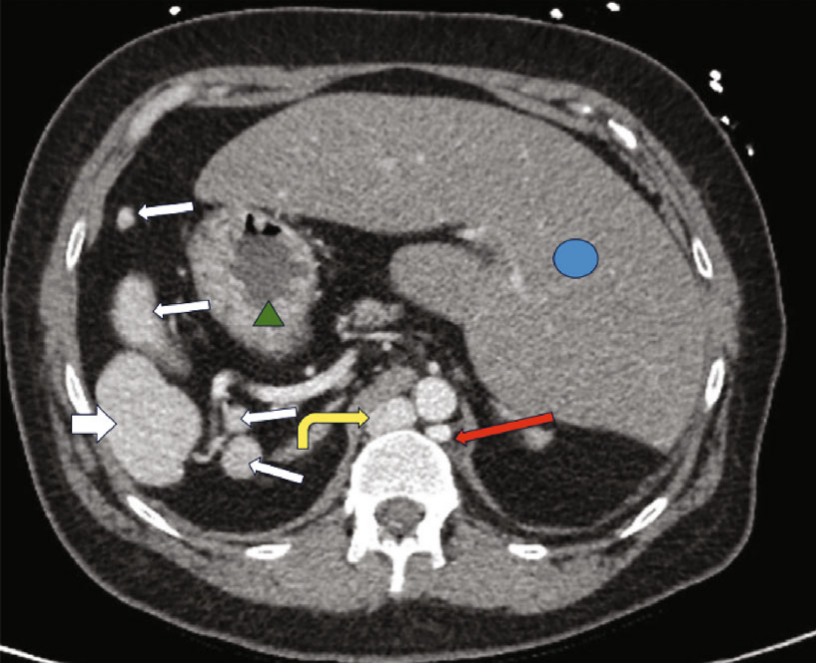
Axial noncontrast CT shows the liver in the LUQ (blue circle), the stomach in the RUQ (green triangle), polysplenia (white arrows), a circumaortic left renal vein (red arrow), and a prominent azygos vein (yellow curved arrow).

A 44-year-old female presented to the emergency department with intermittent abdominal and groin pain, associated with nausea, vomiting, and pain with urination for the past few days. Her medical history was significant for Type 2 diabetes mellitus and chronic lymphocytic leukemia, for which she was under regular surveillance. On physical examination, the patient appeared generally well but reported tenderness on palpation of the right upper quadrant (RUQ) of the abdomen that radiates to her groin during urination. No other significant abnormalities were noted on examination. Given her symptoms and medical history, a comprehensive abdominal imaging study was ordered to further investigate the cause of her abdominal and urinary pain. A noncontrast computed tomography (CT) scan of the abdomen and pelvis revealed a 4 mm obstructive proximal right ureteral calculus as the source of the patient's pain (Figure 1). Notably, there was no history of prior evaluation for situs ambiguous, making this an incidental discovery. Incidentally noted were several anatomical anomalies consistent with situs ambiguous. The liver, gallbladder, and pancreatic head were found to be abnormally positioned in the left upper quadrant (LUQ). Additionally, the stomach was abnormally located in the RUQ. Multiple small splenules without a parent spleen were also seen clustered in the RUQ (Figure 2).

**Figure 3:**
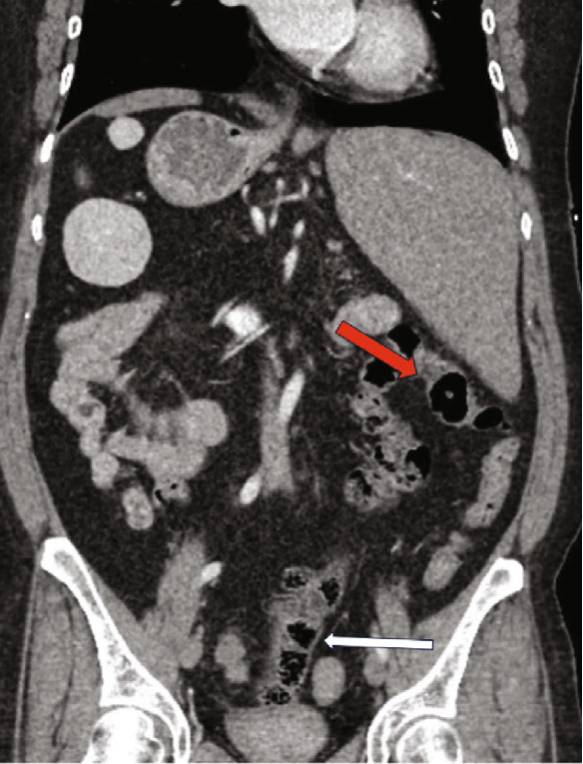
Coronal noncontrast CT demonstrates the midline position of the cecum and ascending colon (white arrow) and a short transverse colon present in the RUQ (red arrow).

The colonic configuration was also abnormal, with the cecum and ascending colon located centrally in the midline, rather than in the expected location of the right hemiabdomen. A short segment of the transverse colon was seen extending from the midline to the left hemiabdomen. The descending and sigmoid colon were normally positioned in the left lower quadrant (LLQ) (Figure 3).

The patient's arterial vasculature was notable for the presence of several variants, including bilateral accessory renal arteries, a common splenogastric trunk, and the common hepatic artery arising directly from the abdominal aorta. Upon evaluation of the venous system, the infrarenal inferior vena cava (IVC) was found to be interrupted with azygous continuation draining to the right heart, an uncommon variant, most frequently associated with polysplenia syndromes [17] (Figure 2). The hepatic veins, however, drained into a separate, normally configured, suprahepatic IVC. A circumaortic left renal vein was also noted (Figure 2).

**Figure 4:**
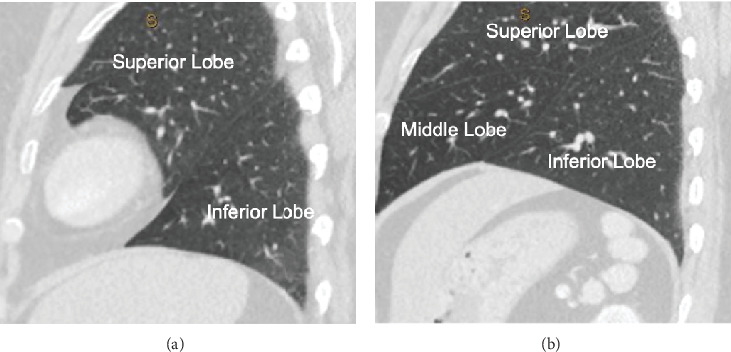
Sagittal CT shows (a) the normal configuration of the bilobed left lung and (b) the normal configuration of the trilobed right lung.

**Figure 5:**
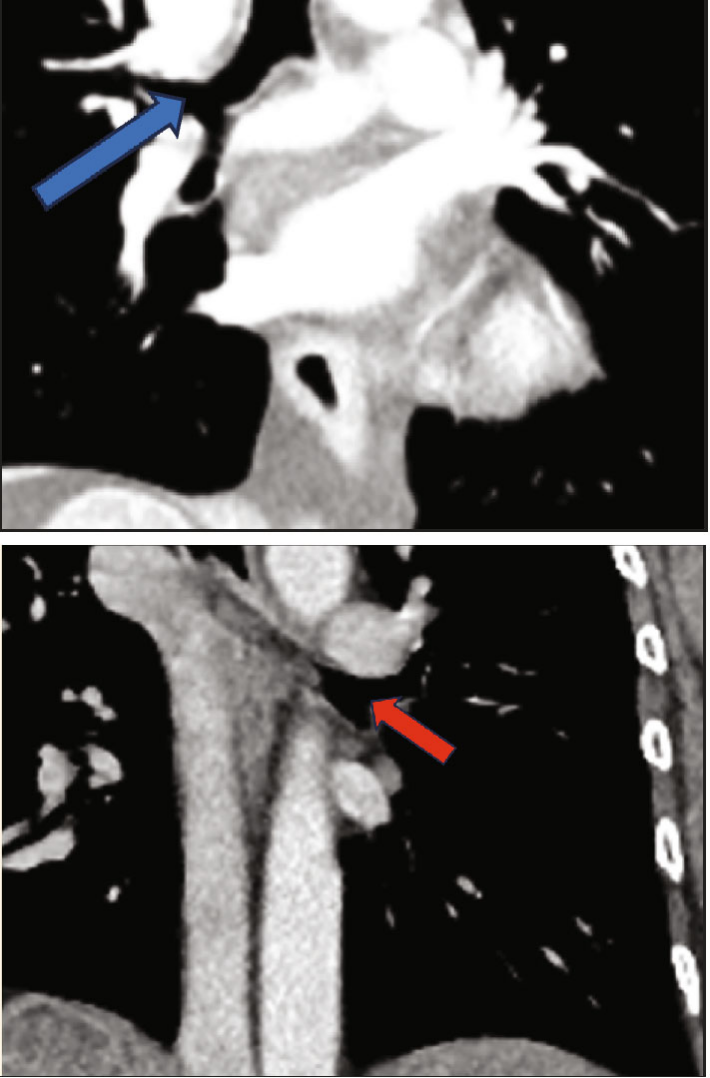
Coronal CT demonstrates normal eparterial right upper lobe bronchus (blue arrow) and hyparterial left upper lobe bronchus (red arrow).

In contrast to the complex abdominal findings, the evaluation of the thorax revealed normal pulmonary anatomy. The lungs were conventionally arranged, with a trilobed right lung and a bilobed left lung (Figure 4). Additionally, there was a normal eparterial right upper lobe bronchus, with the bronchus branching above the level of the ipsilateral pulmonary artery, and a normal hyparterial left upper lobe bronchus, with the bronchus branching below the ipsilateral pulmonary artery (Figure 5).

**Figure 6:**
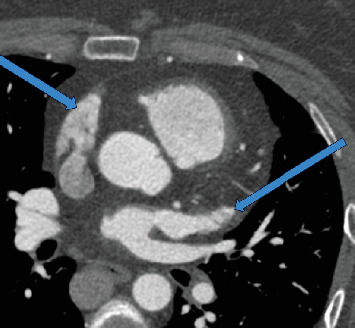
Coronal CTA demonstrates long and narrow bilateral atrial appendages suggesting left isomerism (blue arrows).

In agreement with a previously performed cardiac computed tomography angiogram (CTA), the bilateral atrial appendages were noted to be long and narrow, both having the morphological appearance of a normal left atrial appendage (Figure 6). There was no evidence of a septal defect or other congenital heart defect by CTA.

During the patient's hospital admission, the right ureteral calculus failed to pass with initial conservative management. Subsequently, the patient underwent ureteroscopy, laser lithotripsy, stone extraction, and right ureteral stent placement. These procedures were successful, leading to the resolution of her symptoms and an uneventful recovery.

## 3. Discussion

The patient's presentation of polysplenia and left atrial appendage isomerism with normal bronchopulmonary anatomy suggests a complex disruption in the L-R symmetry signaling pathways, potentially involving the Nodal, LEFTY, and BMP signaling pathways.

Nodal signaling is crucial for establishing the L-R asymmetry, typically on the left side of the embryo, and is regulated by antagonists such as LEFTY1 and LEFTY2 to prevent signal transgression across the midline. Disruptions in the Nodal signaling pathway can lead to a spectrum of laterality defects, from isolated cardiovascular anomalies to complex heterotaxy syndromes [18–20]. No genetic evaluation study was performed in this patient, limiting the ability to determine specific genetic contributions to her unique presentation. Our patient's phenotype indicated selective disruption, possibly due to a mutation or variation in the Nodal pathway that affected abdominal and cardiac organogenesis while sparing pulmonary structures.

BMP signaling modulates Nodal activity through the induction of LEFTY1 in the midline. Disruptions in BMP receptor function have been associated with left isomerism, suggesting a critical role in early L-R axis specification [18]. The normal pulmonary anatomy in this patient implied that Nodal signaling might have been selectively active in the thoracic region, while BMP-mediated inhibition of Nodal signaling in the abdominal region was compromised.

The transcription factor PITX2, which is downstream of Nodal and LEFTY, is important to specify for visceral organ asymmetry. Some cardiac anomalies can be explained through selective disruption of PITX2 function with the lungs being spared, suggesting a mutation that downregulated PITX2 specifically in the cardiac region [16, 21]. Genetic testing targeting PITX2 genes and other related genes may have helped shed more light on the molecular pathways involved in our patient's condition. A further understanding of these mechanisms may eventually allow for more targeted management strategies and contribute to our knowledge about the role of PITX2 and other genes in L-R asymmetry [16, 20].

Moreover, investigating the function of motile cilia in the L-R asymmetry pathway may also provide further insight. The motile cilia have a significant role in creating initial symmetry by forcing fluid toward the left side of the embryo. Mutations that affect ciliary mobility might contribute to the selective preservation of normal pulmonary anatomy, while at the same time disrupting other systems. Further understanding of the patient's anatomical presentation could therefore be provided by exploring ciliary gene mutations and defects.

Lastly, it is important to look into how genetic and environmental factors worked together during this patient's development. Genetic changes can be either modified or worsened by environmental influences during embryogenesis, which underlie these observed unique expressions. Therefore, mothers' health and exposure to teratogens could be important for understanding environmental agents that modulate phenotypic expression of L-R asymmetry disorders more precisely.

## 4. Conclusion

Our case of a 44-year-old female with situs ambiguous, polysplenia, and left atrial appendage isomerism, coupled with normal bronchopulmonary anatomy, emphasizes the variability of anatomic presentations associated with left isomerism. The dissociation between thoracic and abdominal isomerism observed in this patient highlights the complexity of this congenital condition and the need for careful diagnostic evaluation. Clinicians should always be on the lookout for these atypical forms of presentation because they may have significant clinical management consequences as well as patient outcomes. In conclusion, future research should focus on embryological and molecular mechanisms that can explain variations in this L-R asymmetry, thus giving more understanding of their pathogenesis toward a more personalized therapeutic approach.

## Data Availability

The data supporting the findings in this case report are available upon request from the corresponding author.
